# Effectiveness  of a Disease Management Program (DMP) in controlling the progression of Chronic Kidney Disease among hypertensives and diabetics.

**DOI:** 10.12688/f1000research.123787.2

**Published:** 2023-03-01

**Authors:** Leena Sequira, Ravindra Prabhu A., Shreemathi S Mayya, Shankar Prasad Nagaraju, Baby S Nayak

**Affiliations:** 1 Medical Surgical Nursing, Manipal College of Nursing, Manipal Academy of Higher Education, Manipal, Karnataka, 576104, India; 2 Nephrology, Kasturba Medical College, Manipal. Manipal Academy of Higher Education, Manipal, Karnataka, 576104, India; 3 Data Science, Manipal Academy of Higher Education, Manipal, Karnataka, 576104, India; 4 Nephrolgy, Kasturba Medical College, Manipal. Manipal Academy of Higher Education, Manipal, Karnataka, 576104, India; 5 Child Health Nursing, Manipal College of Nursing. Manipal Academy of Higher Education, Manipal, Karnataka, 576104, India

**Keywords:** Hypertension, Type 2 DM, Disease Management Program, estimated Glomerular Filtration Rate, Chronic Kidney Disease

## Abstract

Background: In India, the number of patients with type II diabetes mellitus in 2006 was 40.9 million and is expected to increase by 2025 to 69.9 million. Annually 1,00,000 new patients get diagnosed with End-Stage Renal Disease and require maintenance dialysis. Diabetes Mellitus and hypertension were the usual triggers of Chronic Kidney Disease (CKD). A structured education program helps in the prevention of diabetes and hypertension related complications.

Methods: This quasi-experimental study was conducted among 88 participants who had hypertension, diabetes mellitus, or both for five or more years with an objective to find the effect of a Disease Management Program on delaying progression of CKD in patients with hypertension or diabetes mellitus.

The baseline data were collected on demographic proforma, serum creatinine, blood pressure, and random blood sugar, and the patients were taught the management of hypertension and diabetes mellitus. In the fourth and the eighth month, blood pressure and blood sugar were reassessed. At one-year blood pressure, blood sugar, and serum creatinine were tested. Baseline and one-year follow-up blood pressure, blood sugar, and estimated Glomerular Filtration Rate were compared. Descriptive statistics and “Wilcoxon signed-rank test” were used to analyze the data.

Results: In one year, the mean systolic blood pressure reduced by six mm of Hg and mean blood sugar by 24 mg/dl. The prevalence of CKD stage three and above (< 60 ml/min/m2) was nine (10.22%). The median decline in eGFR was 5 ml/min/m2 (Z= 5.925, P< 0.001).

Conclusion: The Disease Management Program led to improvements in blood pressure and diabetes control and median progression of CKD was estimated at five ml/min/m2/year.

## Introduction

Non-Communicable Diseases (NCD) are the most remarkable cause of fatality in the world. The global predicted prevalence of diabetes among adults is 439 million by 2030 ( Shaw, Sicree, & Zimmet, 2010). The estimated global prevalence of diabetes in 2019 is 9.3% expected to rise to 10.2% by 2030 ( Saeedi *et al.*, 2019). Stage 3 Chronic Kidney Disease (CKD) in people with diabetes is reported to be high (56%) in Cambodia ( Thomas, van, Mehrotra, Robinson-Cohen, & LoGerfo, 2014) ^.^ CKD was 10·8% in rural areas of China. Hypertension and diabetes were associated with CKD ( Zhang *et al.*, 2012). In the United States, 23.5% of individuals aged above 18 years, had CKD ( McFarlane *et al.*, 2011).

The studies conducted in India, on the prevalence of CKD especially among people with hypertension and type 2 DM. The occurrence of diabetes in adults has increased in India ( Tandon *et al.*, 2018). The prevalence of diabetes mellitus was 8.3%, with only 18% receiving treatment ( Tripathy *et al.*, 2017). Type 2 DM and hypertension were the usual triggers of CKD ( Rajapurkar *et al.*, 2012). Hyperglycemia is causing an increase of CKD cases in India. Programs are needed to reduce the risk factors of diabetes mellitus ( Tripathy, 2018). CKD was found in 34.91% of the general population, aged above 18 years, from Varanasi, India ( Rai, Jindal, Rai, Rai, & Rai, 2014). Screening programs are needed to identify CKD in a risk group ( Ene-Iordache *et al.*, 2016). The occurrence rate of stage 5 CKD was 151 per million population ( Modi & Jha, 2006). Lack of knowledge about CKD was observed in people with diabetes ( Fiseha & Tamir, 2020). Diabetes and hypertension were associated with low eGFR and proteinuria ( Singh *et al.*, 2009).

Screening of high-risk populations for CKD helps in the initial detection of CKD ( Bradshaw *et al.*, 2019). It is observed that people are unaware of the complications of diabetes and hypertension. Educating the people is essential before they could land up with CKD ( Hussain, Habib, & Najmi, 2019). The CKD was found to be 24.2% among people aged above 50 years in rural Pondicherry, India. The study suggests targeted screening of adults to prevent further progression of CKD ( Kumar, Dongre, Muruganandham, Deshmukh, & Rajagovindan, 2019). A structured education program helps in the prevention of diabetes-related complications ( Iqbal & Heller, 2018).

The National kidney foundation has defined five stages in CKD, and in the fifth stage, a patient needs dialysis or kidney replacement to live. GFR can be estimated by using the Chronic Kidney Disease – Epidemiology Collaboration (CKD - EPI) formula ( Michels *et al.*, 2010). Serum creatinine is widely used to measure the eGFR ( Coresh *et al.*, 2002). Detection of CKD at the beginning stages helps to slow down progress, which in turn reduces the financial load on individuals, families, and communities.

Studies suggest that the prevalence of hypertension and diabetes is increasing in India and it is the main cause of CKD. There is a clear lack of knowledge about the risks associated with uncontrolled diabetes and hypertension. Most of the population are diagnosed with type 2 DM but are not rightly educated about the complications of negligence associated with it. So, this lack of knowledge has been identified as one of the major reasons for the progress of CKD. Hence the present study intends to look at this aspect and educate the population about the same and monitor their progress across one year period.

## Methods

### Study design and participants

A quantitative approach with quasi-experimental, one group pretest- posttest design was used in this study. The aim of this study was to find the effect of a Disease Management Program (DMP) on delaying progression of CKD in patients with hypertension or type 2 diabetes mellitus.

The participants were the people diagnosed with hypertension and/or diabetes for five or more year's duration and treatment. People visiting rural health centers of Udupi District, Karnataka State, India, aged 30 years and above were the sampled population selected through enumerative sampling technique. Sample size calculated to reach statistical significance with a power of 0.8, a standard deviation of eight, decline in eGFR in one year of five, and significance level 0.05, the total sample required was 22 each in stages one, two, and three of CKD. Keeping a 5% nonresponse rate total sample estimated was 70. The Chronic Kidney Disease stage was known after the serum creatinine test and formula application; hence the total sample taken was 103. Out of 103, for one year, 15 participants failed to follow up and hence 88 samples were analyzed.

### Study instruments

The data were collected using demographic proforma which includes, age, gender, height, weight, serum creatinine, blood pressure, RBS, hypertension, and diabetes mellitus status, and duration of illness. A calibrated weighing scale was used to measure the weight. New measuring tape, sphygmomanometer, and glucometer were used to assess the height, blood pressure, and blood sugar, respectively. For checking blood pressure calibrated sphygmomanometer with appropriate cuff size of 13.1 × 23.5 cm was used. Before blood pressure measurement was taken it was made sure that the samples has not consumed tea or coffee, smoked or exercised vigorously in the last 30 minutes. Blood pressure was measured in the sitting position on the upper arm with the arm supported, and sphygmomanometer at the level of the heart.The investigator checked blood pressure twice in one sitting and average of the two readings recorded. The intervention, DMP, refers to educating the participants about the management of hypertension or diabetes mellitus on a one-to-one basis (explaining and giving leaflets) and follows up on every fourth month, till one year, with teaching reinforcement along with random blood sugar and blood pressure assessment, as well.

Development of the education module and leaflet about hypertension and diabetes mellitus was prepared by the researcher by reviewing the published and unpublished literature and validated by experts. The education module contains the meaning, causes and risk factors, signs and symptoms, diagnosis, and management. Management included nutrition, exercise, monitoring of blood sugar and blood pressure, pharmacologic therapy. An explanation about the disease is given in a simple, understandable way, and doubts raised by patients were cleared. Complications were explained to improve the compliance level. The importance of exercise, nutrition, and compliance with medication in controlling blood sugar and blood pressure were also explained.

The researcher filled the demographic proforma by collecting information from the participants and assessed height, weight, blood pressure, and Random Blood Sugar **(**RBS). Blood for serum creatinine was collected using serum vacutainer and assessed using the standard Jaffe method calibrated to Isotope Dilution Mass Spectrometry (IDMS). CKD-EPI formula was used to estimate GFR. Teaching was given about managing hypertension and diabetes mellitus, and a leaflet about the same was distributed during the baseline data collection. Fourth and eighth-month blood pressure and RBS were reassessed, and teaching was reinforced. At one-year blood pressure, RBS, and serum creatinine were tested.

### Study variables

Demographic variables were age, gender, serum creatinine height, and weight. Teaching regarding management of Hypertension and Diabetes Mellitus is the independent variable. Blood pressure and RBS were the dependent variables that affect kidney function and eGFR is the key variable. Other variables are disease conditions (Diabetes Mellitus or Hypertension or both) and duration of illness.

### Data analysis

Data were analyzed using SPSS. Continuous variables are summarized using mean or median whichever is applicable and categorical variables using proportions. Frequency and percentage were used to describe the participant characteristics. Blood pressure and RBS were the dependent variables that affect kidney function. Mean, standard deviation, and range were used to summarize blood pressure, RBS, and paired’t’ test to compare baseline and at one year follow up systolic blood pressure (SBP), diastolic blood pressure (DBP), and RBS. As per, kidney disease: Improving Global Outcomes (KDIGO) Guidelines CKD is classified into five stages. Stage 1 (GFR ≥ 90 ml/min), stage 2 (GFR = 60-89 ml/min), Stage 3 (GFR = 30-59 ml/min), Stage 4 (GFR = 15-29 ml/min), stage 5 (GFR < 15 ml/min). Cross table was used to explain the number of participants who improved, remained in the same stage of CKD, and progressed to a higher stage of CKD. “Wilcoxon signed-rank test” was used to find the effectiveness of DMP as data (eGFR) were not following normality. The difference between baseline and one-year follow-up GFR is done and categorized into ≤1 ml, 1-10 ml, and more than 10 ml.

### Ethical considerations

The study protocol was approved by the Kasturba Medical College and Kasturba Hospital Institutional Ethics Committee. (IEC184/2011). The participant information sheet was given to the participants, and the study process was explained and informed written consent was obtained from the participants before data collection.

## Results

### Demographic characteristics of participants

Demographic characteristics of baseline and one-year follow-up are summarized in Table 1. About 87.5 % of them belong to the age group of 51 years, and above, 46.6% of them were hypertensive, and 35.2% of them had both hypertension and type 2 DM.

**Table 1.** Sample characteristics in frequency and percentage.

Variables		Baseline N = 103	At one year N = 88
		f (%)	f (%)
Age (in years)	30-40	2 (1.9)	2 (2.3)
	41-50	14 (13.6)	9 (10.2)
	51 & above	87 (84.5)	77 (87.5)
Mean age		61 ± 10.7	63 ± 10.5
Gender	Male	52 (50.5)	45 (51.1)
	Female	51 (49.5)	43 (48.9)
Disease status	Hypertension	49 (47.6)	41 (46.6)
	Diabetes Mellitus (DM)	20 (19.4)	16 (18.2)
	Both Hypertension &DM	34 (33)	31 (35.2)
Serum creatinine (mg/dl)	≤1.5	100 (97.10)	82 (93.18)
	>1.5-1.9	3 (2.90)	6 (6.82)
Mean ± SD		0.916 ± 0.24	1.07 ± 0.30
Hypertension duration (in years)	≤10	63 (75.90)	53 (73.60)
	11-15	11 (13.25)	10 (13.90)
	>15	9 (10.85)	9 (12.50)
Diabetes duration (in years)	≤10	42 (77.78)	35 (74.47)
	11-15	5 (09.26)	5 (10.64)
	>15	7 (12.26)	7 (14.89)

### Mean, standard deviation and range of BP and RBS and comparison of SBP, DBP & RBS using paired t' test

Table 2 shows mean, standard deviation, range of blood pressure, and RBS at the four-month interval and results of paired t' test applied to compare baseline and at one year follow up systolic blood pressure (SBP), diastolic blood pressure (DBP) and RBS. At baseline, most (69.44%) of them had SBP of 141-220 mm of Hg, 29.78% of them had an RBS level of 201-400 mg/dl. Mean SBP reduced by 6 mm of Hg and mean RBS by 24 mg/dl at one year, follow-up. There was a significant reduction in blood pressure and RBS (p < 0.001) for one-year follow-up.

**Table 2.** Mean, standard deviation and range of BP and RBS and comparison of SBP, DBP & RBS using paired‘t’ test.

N=88						
	Mean & SD SBP	Mean & SD DBP	Mean & SD RBS	Range SBP	Range DBP	Range RBS
**Baseline**	144 (21)	86 (9)	184 (85)	110-220	60-110	89-480
**At four months**	140 (15)	85 (7)	170 (53)	110-180	70-100	92-360
**At eight months**	138 (13)	84 (6)	162 (38)	110-176	70-98	96-258
**At one year**	138 (14)	85 (7)	160 (38)	110-180	60-100	100-252
**‘t’ Value**	3.409	1.731	2.840			
**‘P’ value**	0.001	0.08	0.007			

### Baseline and one-year follow-up stages of CKD

Table 3 shows the baseline and one-year follow-up stages of CKD. At baseline, 47 participants had stage 2 CKD. Among them, four of them improved to stage 1, and 13 of them progressed to stage 3 CKD. At baseline, eight participants had CKD stage 3. Out of eight, two of them improved to stage 2, and one progressed to stage 4 CKD, and five remained in the same stage.

**Table 3.** CKD stages at baseline and one year follow up using CKD - EPI formula.

N= 88						
CKD stages – At one year follow up						
**CKD – stages baseline**		**1**	**2**	**3**	**4**	**Total**
	**1**	**13 (40.60)**	19 (59.40)	0	0	32 (100)
	**2**	4 (8.5)	**30 (63.8)**	13 (27.7)	0	47 (100)
	**3**	0	2 (25)	**5 (62.5)**	1 (12.50)	8 (100)
	**4**	0	0	0	**1 (100)**	1 (100)
	**Total**	17 (19.3)	51 (58)	18 (20.40)	2 (2.3)	88 (100)

Values in the parenthesis are row percentages. This is cross table; row total represents the baseline and column total represents the one year follow up stages of CKD.

### Effectiveness of the DMP

Table 4 shows the effectiveness of the DMP. The pre and post-intervention eGFR data of participants was not following normality, hence median, median difference, and 'Z' value of pre and post-intervention eGFR were assessed. The median fall in GFR is 5 ml/min/m ^2^/year and there is a significant difference in GFR change in one year follow up, which says the intervention is not effective. The intervention helped to delay renal function deterioration. Table 5 shows the progression of CKD for a one-year follow-up. About 36.4% of participants lost only less than 1ml of GFR for one year.

**Table 4.** Median, median difference, and ‘Z’ value of pre and post intervention eGFR.

N = 88			
	N	Median	IQR
Pre (Baseline)	88	83	24
Post (After 1 year	88	78	26

Wilcoxon Signed Ranks Test z= 5.925, P < 0.001.

**Table 5.** Progression of CKD for one year follow up.

eGFR loss (ml)	DM & Hypertension N = 31	DM N = 16	Hypertension N = 41	Hypertension, DM and Both (41+16+31) N = 88
≤1 ml	14 (45.2)	7 (43.8)	11(26.8)	32 (36.4)
1-10 ml	5 (16.1)	2 (12.5)	12 (29.3)	19 (21.6)
>10 ml	12 (38.7)	7 (43.8)	18 (43.9)	37 (42)

## Discussion

The result of the present study shows that CKD stage 3 and higher amounts to 10.22% of the participants. There have been few large community-based studies looking at the prevalence of CKD among hypertensive and diabetic populations in India and other countries. A study done in India reported that among 6129 participants, 2578 are having hypertension and CKD is present in 23.5% of hypertensive patients ( Farag *et al.*, 2014). Another study done in China among 1039 patients diagnosed with type 2 DM aged over 30 years shows 32.8% of CKD stage 3-5 ( Lu *et al.*, 2008). Collectively the reflection indicates that Type 2 DM and hypertension are the important public health issues, and it is associated with kidney disease.

The present study shows, in a year, mean systolic blood pressure decreased by 6 mm of Hg. A few participants confided that they were skipping the medication as signs and symptoms of the disease were not evident, but due to DMP, they returned to regular medication. Studies done in other countries suggested that DMP and assessing blood pressure, blood sugar, and GFR helps to sensitize the patients about their disease condition and to seek nephrology references if needed. DMP leads to improved hypertension control and eGFR among participants with CKD stage four or five ( Richards *et al.*, 2008). A study done in India by kidney help to screen the entire population of one village and provide medication for hypertension and diabetes showed a decrease in the prevalence of CKD ( Prabahar, Chandrasekaran, & Soundararajan, 2008). Another study done in Australia among patients with diabetes, CKD, and hypertension showed no significant improvement in the intervention group (n = 36) in terms of medication adherence and blood pressure control. However, there was a 6 mm Hg reduction in SBP ( Williams, Manias, Walker, & Gorelik, 2012b). Another study showed no significant differences in drug adherence between the intervention and control groups ( Williams, Manias, Liew, Gock, & Gorelik, 2012a). The study on the impact of eGFR reporting on referral rates shows that eGFR reporting was useful in reducing the late referral to nephrology services ( Foote *et al.*, 2014). Hence it is necessary to identify the early stages of CKD, educate them about the importance of disease management.

The present study reports that the rate of drop in eGFR in one year was 5 ml/minute/1.73 m ^2^. At one year follow-up, 36.4% lost less than 1 ml, and 21.6% lost 1-10 ml of eGFR. However, there are no research studies done in India to compare the change in eGFR in one year. The fall in eGFR ranges from 2-20 ml/minute/1.73 m ^2^/year ( Snyder & Pendergraph, 2005). In a study done in the US among people with diabetes, the eGFR dropped at 2.8 ml/min/1.73 m ^2^ per year ( Hanratty *et al.*, 2010). A study done among the Rural Diabetic Cambodian population shows, at a median of 433 days follow up, 32% of patients lost more than or equal to 5 ml/min/m ^2^ of eGFR ( Thomas, Pelt, Mehrotra, Robinson-Cohen, & LoGerfo, 2014). Further studies are required to find the rate of decline in GFR in normal individuals and individuals with comorbidities.

### Study limitation

Glomerular Filtration Rate was estimated and not measured. The control group was not used due to ethical reasons. Only patients with diabetes and hypertension aged 30 years and above, were studied.

## Conclusion

The Disease Management Program led to improvements in blood pressure and diabetes control and median progression of CKD was estimated at 5 ml/min/m ^2^/year. Regular assessment of eGFR of the risk group, sensitizes the patient about their renal function. Teaching about the management of hypertension and diabetes mellitus and checking blood pressure and RBS helps to know about their disease control and to take action to control blood pressure and blood sugar.

### Implications to nursing practice, management, policy education and future research

People in the community are unaware of the seriousness of CKD. In the prevention and control of CKD, nurses can play an important role. In the outpatient department, nurses can educate the patients with hypertension and diabetes mellitus, and thus implement an effective DMP at the early stages of CKD. The nurse can work in the field with peripheral clinic workers for monitoring and evaluating each person by checking blood pressure and blood sugar and screening for CKD.

## Data availability

Figshare: Progression of chronic kidney disease in patients with hypertension or type 2 diabetes mellitus, can it be delayed? DOI: https://doi.org/10.6084/m9.figshare.20278266

The project contains the following underlying data:

- Data file 1 (Sample size calculation)
- Data file 2 (Education program handout)
- Data file 3 (Raw Data)

Data are available under the terms of the Creative Commons Attribution 4.0 international licence (CC BY 4.0)
