# Supplementary material for: Effectiveness  of a Disease Management Program (DMP) in controlling the progression of Chronic Kidney Disease among hypertensives and diabetics
Source: F1000Res. 2024 Mar 12;11:1111. Originally published 2022 Sep 28. [Version 3] doi: 10.12688/f1000research.123787.3 (PMC10993008; doi:10.12688/f1000research.123787.3)
Supplement: Peer review history

# Reviewer report: Reviewer response for version 3

**DOI:** 10.5256/f1000research.163293.r255014

**Published:** 2024-04-03

**Authors:** Ashwani Kumar

**Affiliations:** 1 The George Institute for Global Health & University of New South Wales, Sydney,, Australia

All my comments have been addressed in the revised manuscript.

Is the work clearly and accurately presented and does it cite the current literature?

Yes

If applicable, is the statistical analysis and its interpretation appropriate?

Partly

Are all the source data underlying the results available to ensure full reproducibility?

Yes

Is the study design appropriate and is the work technically sound?

Yes

Are the conclusions drawn adequately supported by the results?

Partly

Are sufficient details of methods and analysis provided to allow replication by others?

Partly

Reviewer Expertise:

Diabetes, critical care, sepsis

I confirm that I have read this submission and believe that I have an appropriate level of expertise to confirm that it is of an acceptable scientific standard.

# Reviewer report: Reviewer response for version 2

**DOI:** 10.5256/f1000research.144890.r236892

**Published:** 2024-02-23

**Authors:** Ashwani Kumar

**Affiliations:** 1 The George Institute for Global Health & University of New South Wales, Sydney,, Australia

General:

 - Language is not scientific at some places.

 - Readability and flow need a lot of improvement as currently it is hard to read.

 - Relevant references are missing at a few places.

 - Abbreviations should be expanded at the first instance.

 Abstract: 

 - Objective is missing. 

 - Conclusion is contradicting the results.

 Introduction:

 - Authors should provide supporting data on Disease Management Plan (DMP) to improve clinical outcomes in order to strengthen the study rationale.

 Methods:

 - Sample size calculation is not clearly articulated.

 - Authors should provide supporting references used to develop DMP.

 - Authors should add details about how follow up was done.

 - Specify who delivered the DMP in the study and if they were trained to do so.

 - The SPSS version needs to be specified.

 Results:

 - Tables headings should be relevant to the presented data

 - Provide relevant percentages with the numbers.

 - Table 4: No Z value and p value is shown

 Discussion: 

 - The first paragraph repeats the information in the Introduction section. Here, authors should summarise the key results. 

 - Limitations should be more detailed and add strengths, if any.

 Conclusion: Implications should be moved to the Discussion section.

Is the work clearly and accurately presented and does it cite the current literature?

Yes

If applicable, is the statistical analysis and its interpretation appropriate?

Partly

Are all the source data underlying the results available to ensure full reproducibility?

Yes

Is the study design appropriate and is the work technically sound?

Yes

Are the conclusions drawn adequately supported by the results?

Partly

Are sufficient details of methods and analysis provided to allow replication by others?

Partly

Reviewer Expertise:

Diabetes, critical care, sepsis

I confirm that I have read this submission and believe that I have an appropriate level of expertise to confirm that it is of an acceptable scientific standard, however I have significant reservations, as outlined above.

# Reviewer report: Reviewer response for version 2

**DOI:** 10.5256/f1000research.144890.r165070

**Published:** 2023-03-24

**Authors:** Manohar Bairy

**Affiliations:** 1 Department of Renal Medicine, Tan Tock Seng Hospital, Singapore City, Singapore

Responses and amendments by the author are noted with thanks.

Is the work clearly and accurately presented and does it cite the current literature?

Partly

If applicable, is the statistical analysis and its interpretation appropriate?

Partly

Are all the source data underlying the results available to ensure full reproducibility?

No

Is the study design appropriate and is the work technically sound?

No

Are the conclusions drawn adequately supported by the results?

No

Are sufficient details of methods and analysis provided to allow replication by others?

No

Reviewer Expertise:

eGFR, CKD, Dialysis

I confirm that I have read this submission and believe that I have an appropriate level of expertise to confirm that it is of an acceptable scientific standard.

# Reviewer report: Reviewer response for version 1

**DOI:** 10.5256/f1000research.135925.r153673

**Published:** 2022-10-27

**Authors:** Manohar Bairy

**Affiliations:** 1 Department of Renal Medicine, Tan Tock Seng Hospital, Singapore City, Singapore

Thanks for asking me to review.

 This is an evaluation of an educational program implemented in a population with diabetes and hypertension with the objective to delay CKD progression.

 I have the following comments to make:

1. Can the research question be more clearly elucidated? More specifically, was the study looking at whether the DMP impacted the eGFR change or whether the DMP impacted the BP and RBS? The assignment of BP and RBS as dependent variables suggests the latter was the study research hypothesis but the title suggests otherwise.
2. The abstract says that 100000 patients enter ESRD per year. Does this refer to the Indian population or the global population? In the second paragraph of the introduction, ' *the occurrence rate of stage 5 CKD*' is mentioned to be 151 per million population in India which amounts to around 200000 entering CKD5. Perhaps these statements could be coalesced and mentioned in the introduction for clarity.
3. The criteria used to diagnose CKD stages 2 and 1 could be clarified as nearly 90% of the participants were in this category. How were they diagnosed to have CKD as eGFR was above 60 - did they have evidence of structural damage (proteinuria, abnormal urine sediment, radiological abnormalities, hereditary diseases.)? As an example, an individual aged 40 with an eGFR of 100 does not have CKD stage 1 unless there is evidence of structural damage. Likewise, an individual with eGFR 78, aged 70 does not have CKD stage 2 unless there is evidence of structural damage. The eGFR is to be used merely for the staging of diagnosed CKD except when the eGFR is <60 when it is used for diagnosing CKD.
4. A discussion of how using RBS by glucometer would compare with using HBA1C or FBS/PPBS as a measure of DM control would be relevant while discussing the strengths and limitations of the study as the RBS is the main dependent variable. Likewise, the method of BP measurement - number of attempts, device used, measured by whom, average of readings in a single sitting - is also worth discussing as the effect size was small (5mm hg of SBP, nil In DBP) and BP was the main dependent variable.
5. I note that the eGFR was merely a key variable rather than the dependent variable. That diminishes the stated intent of the study.
6. Would it be possible to include the eGFR at baseline and at the end of the study in Table 1 as it is not in the data collection sheet?
7. In the results section, in the paragraph headed ' *Effectiveness of the DMP’* the author says: ‘ *The median fall in GFR is 5 ml/min/m2/year and there is a significant difference in GFR change in one year follow up, which says the intervention is not effective. The intervention helped to delay renal function deterioration*.’ - this needs further clarification due to the contradictory nature of the two statements.
8. In the first paragraph of the Discussion section, it is worth commenting on whether the study sampling was considered representative of the population and hence comparable to the other studies referenced.
9. It appears there are missing values for BP – how they were dealt with could be mentioned.
10. Could the fall in BP be attributed to starting antihypertensives during the course of the study or titration of dose as part of the standard of care rather than the DMP? The lack of a control group or historical data (BP, RBS, eGFR) for the same group of patients impacts the precision and validity of the study.
11. In the data collection sheet, the data collection time points mentioned are the 4 ^th^, 6 ^th,^ and 12 ^th^ month. Elsewhere, the 4 ^th^, 8 ^th,^ and 12 ^th^ month is mentioned.
12. Table 3 suggests there was significant progression of CKD through the stages, for example, 15 individuals with stage 1 progressed to stage 2, and 13 progressed from stage 2 to stage 3 within the span of 1 year. eGFR data would help the reader visualise the continuous nature of the data.
13. Overall, it is reasonable to conclude that a DMP program is feasible in a population with DM and hypertension attending rural health centres in India. Qualitative data on the impact of the education program would be useful in the absence of a control group or historical pre-test data.

Is the work clearly and accurately presented and does it cite the current literature?

Partly

If applicable, is the statistical analysis and its interpretation appropriate?

Partly

Are all the source data underlying the results available to ensure full reproducibility?

No

Is the study design appropriate and is the work technically sound?

No

Are the conclusions drawn adequately supported by the results?

No

Are sufficient details of methods and analysis provided to allow replication by others?

No

Reviewer Expertise:

eGFR, CKD, Dialysis

I confirm that I have read this submission and believe that I have an appropriate level of expertise to state that I do not consider it to be of an acceptable scientific standard, for reasons outlined above.

# Response

**Published:** 2023-02-25

**Authors:** leena sequira

**Affiliations:** Manipal Academy of Higher Education, India

Thank you for the detailed review. Please find the response for the comments. 

1. Title is modified - Effectiveness of a Disease Management Program (DMP) in controlling the progression of Chronic Kidney Disease among hypertensives and diabetics.
  Uncontrolled hypertension and diabetes were the main causes of CKD. Hence the researcher studied the people with hypertension and diabetes.
2. The abstract was modified adding this sentence -The occurrence rate of stage 5 CKD was 151 per million population in India.
3. Classification of CKD is done based on eGFR. Other diagnostic measures were not done.
  That is one of the limitations.
4. For checking blood pressure calibrated sphygmomanometer with appropriate cuff size of 13.1 × 23.5 cm was used. Before blood pressure measurement was taken it was made sure that the samples has not consumed tea or coffee, smoked or exercised vigorously in the last 30 minutes. Blood pressure was measured in the sitting position on the upper arm with the arm supported, and sphygmomanometer at the level of the heart. The investigator checked blood pressure twice in one sitting and average of the two readings recorded. The researcher mainly focused on eGFR. Kidney function depends on Control of blood sugar and blood pressure also.
5. eGFR is a dependent variable and Disease management program was the independent variable.
6. Included in the data sheet.
7. Statistically significant change in eGFR was observed. People with hypertension and diabetes, there will be more chances of decrease in eGFR. But intervention helped to delay the progression of GFR. The research design was not strong to prove this. Control group was not included, based on the experts suggestion.
8. In the studies selected for the discussion , the study population was representative of the population.
9. Blood pressure was checked for all patients and entered in data sheet only for patients with history of hypertension.
10. Fall in BP could be due to the education and sensitization of the people about the complications of uncontrolled hypertension.
11. Correction is done in the data sheet.
12. Base line and at one year GFR included in the data sheet.
13. Good suggestion.
